# An Investigation of a Role for U2 snRNP Spliceosomal Components in Regulating Transcription

**DOI:** 10.1371/journal.pone.0016077

**Published:** 2011-01-24

**Authors:** Susannah L. McKay, Tracy L. Johnson

**Affiliations:** Molecular Biology Section, Division of Biological Sciences, University of California San Diego, La Jolla, California, United States of America; University of Edinburgh, United Kingdom

## Abstract

There is mounting evidence to suggest that the synthesis of pre-mRNA transcripts and their subsequent splicing are coordinated events. Previous studies have implicated the mammalian spliceosomal U2 snRNP as having a novel role in stimulating transcriptional elongation *in vitro* through interactions with the elongation factors P-TEFb and Tat-SF1; however, the mechanism remains unknown [1]. These factors are conserved in *Saccharomyces cerevisiae*, a fact that suggests that a similar interaction may occur in yeast to stimulate transcriptional elongation *in vivo*. To address this possibility we have looked for evidence of a role for the yeast Tat-SF1 homolog, Cus2, and the U2 snRNA in regulating transcription. Specifically, we have performed a genetic analysis to look for functional interactions between Cus2 or U2 snRNA and the P-TEFb yeast homologs, the Bur1/2 and Ctk1/2/3 complexes. In addition, we have analyzed Cus2-deleted or -overexpressing cells and U2 snRNA mutant cells to determine if they show transcription-related phenotypes similar to those displayed by the P-TEFb homolog mutants. In no case have we been able to observe phenotypes consistent with a role for either spliceosomal factor in transcription elongation. Furthermore, we did not find evidence for physical interactions between the yeast U2 snRNP factors and the P-TEFb homologs. These results suggest that *in vivo*, *S. cerevisiae* do not exhibit functional or physical interactions similar to those exhibited by their mammalian counterparts *in vitro*. The significance of the difference between our *in vivo* findings and the previously published *in vitro* results remains unclear; however, we discuss the potential importance of other factors, including viral proteins, in mediating the mammalian interactions.

## Introduction

Removal of introns from eukaryotic pre-mRNA is carried out by a large, dynamic macromolecular machine called the spliceosome. Although pre-mRNA splicing was once thought to be a distinct biochemical process, work in the last 10 years has done much to demonstrate that pre-mRNA splicing can occur co-transcriptionally as the pre-mRNA is being transcribed by RNA polymerase II (RNAPII). The temporal and spatial coordination of these processes affords the opportunity for factors involved in each process to influence the other. Indeed, recent work has established that molecular and functional interactions take place between the RNAPII elongation complex and the RNA splicing machinery [2]–[4]. These interactions work to coordinate the two processes with one another in a manner that is thought to ensure efficient production and processing of mRNA. Understanding how these processes are coordinated is crucial for understanding gene expression.

The polymerase carboxyl-terminal domain (CTD) is important for coordinating pre-mRNA splicing and transcription. The CTD has been shown to physically interact with splicing factors and to positively regulate splicing *in vitro* and *in vivo* [4], [5]. Post-translational modifications of the polymerase CTD by kinases, phosphatases, and prolyl isomerases have been shown to affect co-transcriptional splicing through multiple mechanisms (for reviews see [6]–[9]).

The mammalian kinase complex P-TEFb (positive transcription elongation factor b) is an essential regulator of transcription elongation and has multiple roles in coordinating transcription and pre-mRNA processing [10], [11]. P-TEFb, comprising CDK9 and its associated cyclin T1, facilitates release of stalled RNAPII into productive elongation through a variety of mechanisms, including inhibition of transcriptional repressors, recruitment of positive elongation factors, and phosphorylation of the polymerase CTD at Serine 2 of its heptapeptide repeat, a modification associated with productive elongation. These activities are required for recruitment of splicing factors to the site of active transcription and stimulation of co-transcriptional splicing [12]–[15].

The role of P-TEFb at the interface of splicing and transcription was highlighted by an important report in 2001 [1]. Here it was demonstrated that immunoprecipitates of P-TEFb containing the elongation factor Tat-SF1 (Tat stimulatory factor 1) and spliceosomal snRNPs stimulated transcriptional elongation of a human immunodeficiency virus-1 (HIV-1) template. The stimulatory effect was dependent upon the ability of Tat-SF1 to associate with both P-TEFb and the U2 snRNA. This finding suggested a novel role for Tat-SF1 and the U2 snRNP in stimulating transcription. However, the detailed mechanism underlying this stimulatory effect remains unknown, and it is not clear if this interaction occurs *in vivo* in mammalian cells.

The yeast homolog of Tat-SF1, Cus2, has been characterized in yeast as a U2 snRNP-associated splicing factor. Tat-SF1 and *CUS2* share 46% sequence identity, and the proteins each contain two RNA recognition motifs (RRMs), as well as an acidic C-terminal domain [16]. The homology between *CUS2* and Tat-SF1 has raised the intriguing question of whether Cus2 has a role in regulating transcription. Recently it was shown that deletion of *CUS2* reduced influenza RNA synthesis in yeast cells infected with viral ribonucleoprotein complex (vRNP) components [17]. Since Tat-SF1 knockdown in influenza-infected mammalian cells affected formation of vRNP particles, independent of RNA synthesis or processing, these studies raised the possibility that Cus2 could be playing a similar role as a chaperone for viral RNP assembly. This study also raised the question of whether Cus2 is capable of exhibiting a stimulatory effect on transcription similar to that reported for Tat-SF1. And, since the yeast cells in this report were infected with viral components, this study raised the question of whether the effects observed are specific to viral systems or are indicative of a more general role for Cus2 in transcription.

**Figure 1:**
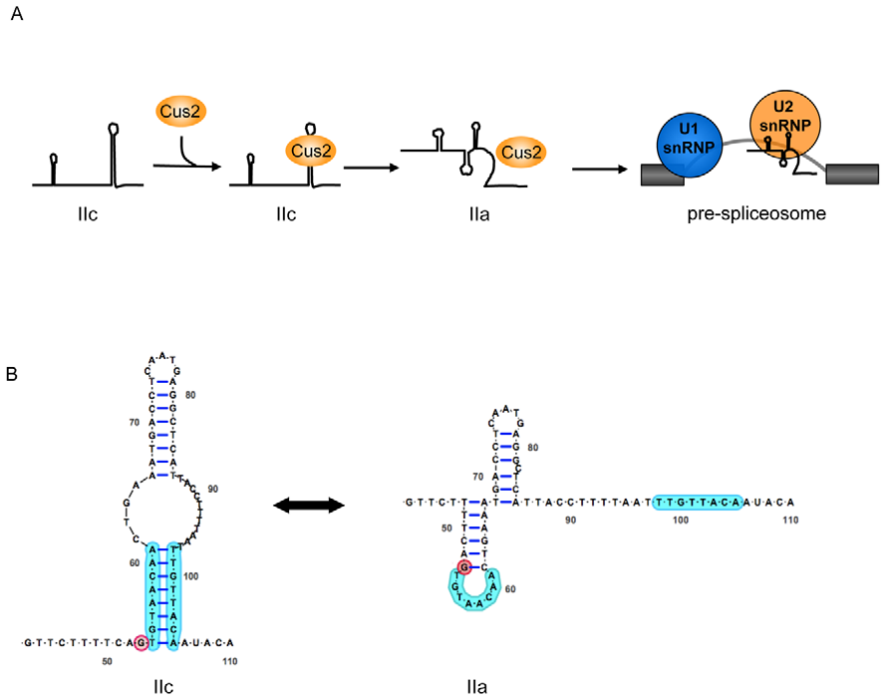
Cus2 facilitates refolding of mutually exclusive structures of the U2 snRNA. (A) Model of Cus2's activity toward the U2 snRNA. Cus2 recognizes and binds the U2-IIc snRNA conformation and facilitates its rearrangement into the U2-IIa conformation. Formation of the U2-IIa conformation activates the snRNP (depicted as a ball) and allows for its stable association with the pre-mRNA substrate to form the pre-spliceosome with the U1 snRNP. The exons of the pre-mRNA are depicted as filled boxes and the intron is depicted as a line. (B) Diagram of the 5′ portion of yeast U2 snRNA mutually exclusive U2-IIc and U2-IIa conformations. The U2 snRNA mutants used in this study promote either U2-IIc or U2-IIa as indicated. The G53 position is shaded red. The sequences that base pair to form the U2-IIc stem are shaded in blue.

Although a direct role for Tat-SF1 in regulating splicing is yet to be determined, the role for *CUS2* in splicing has been well characterized. The U2 snRNA of yeast and humans is very similar, with the exception of a unique, 945 nucleotide fungal stem loop which is dispensable for the RNA's role in splicing [18]. The highly conserved 5′ end of the U2 snRNA can adopt multiple secondary structures at several steps during the splicing cycle [19]–[26]. Regulated formation of these structures is required for both spliceosome assembly and the catalysis of splicing. During spliceosome assembly, Cus2 recognizes and binds the IIc conformation of the U2 snRNA and, along with the helicase Prp5, facilitates its refolding into the IIa conformation. Formation of the U2-IIa structure activates the snRNP and allows for its stable association with the pre-mRNA [19], [24]–[26] (see also Figure 1). The ability of Cus2 to facilitate U2 snRNA folding is abrogated by a mutation within one of its RRMs that abolishes U2 snRNA binding *in vitro* [16]. A corresponding mutation in Tat-SF1 has been shown to disrupt the protein-snRNP interaction necessary to enhance elongation. This raises the possibility that the ability of Tat-SF1 to associate with the U2 snRNP, perhaps mediated by the snRNA's secondary structure, is responsible for the effects on elongation [1]. Notably, in mammals the U1 snRNA has been shown to stimulate transcription initiation by TFIIH, a result which suggests a precedent for spliceosomal snRNAs in affecting transcriptional events [27].

In addition to the homology between *CUS2* and Tat-SF1, *S. cerevisiae* also possess factors homologous to P-TEFb. The cyclin-dependent kinases (CDKs) Bur and Ctk exhibit equivalent levels of sequence homology with P-TEFb [28] and, like P-TEFb, are important for regulating several aspects of transcription. Hence, it has been proposed that together the Bur and Ctk complexes reconstitute the activity of their mammalian counterpart [10], [29]. However, a detailed functional analysis of several metazoan CDKs suggest that Bur1 and its associated cyclin Bur2 are orthologous to the metazoan Cdk9/cyclin T1, while Ctk1 and its cyclin are actually orthologs of the metazoan Ctk12/cyclin K [30]. The yeast Bur and Ctk complexes appear to facilitate transcriptional elongation at the 5′ and 3′ ends of genes, respectively, through a variety of mechanisms including CTD phosphorylation and chromatin modification (for review see [29]). The Bur1 kinase is required for efficient elongation by the polymerase and phosphorylates the CTD of RNAPII at Serine 2 near the promoter of genes [31] and at Serine 7 [32]. The Bur complex also acts by targeting non-CTD substrates to regulate histone modification [33], [34] and to suppress cryptic transcription [35]. The Ctk complex consisting of the Ctk1 kinase, its associated Ctk2 cyclin, and a third regulatory protein, Ctk3, is also required for elongation by the polymerase, phosphorylates Serine 2 of the CTD, and regulates histone H3 trimethylation [36]–[39].

Strong sequence conservation of P-TEFb, Tat-SF1, and the U2 snRNA from mammals to simpler eukaryotes supports the idea that the mechanism whereby P-TEFb and Tat-SF1/U2 snRNA interact to affect elongation may be conserved throughout evolution. To address this possibility, we analyzed the relationship of the homologous factors in yeast. We hypothesized that, if a similar interaction exists in yeast, disruption of either *CUS2* or the U2 snRNA would result in the same transcription-related phenotypes exhibited by perturbation of the CDKs. Furthermore, we predicted that these factors would physically associate in a manner similar to their mammalian counterparts.

We performed a genetic analysis in *Saccharomyces cerevisiae* to investigate the *in vivo* relationship of the CDKs and the U2 snRNP components, Cus2 and U2 snRNA. We find that neither mutating *CUS2* nor the U2 snRNA exhibit genetic interactions with the Bur or Ctk complexes. In addition, mutations of the U2 snRNP components do not exhibit phenotypes that have been used previously to assess the roles of P-TEFb homologs in transcription; these phenotypes include sensitivity to 6-azauracil (6-AU), inositol auxotrophy, and the Spt^−^ or Bur^−^ phenotypes. Finally, we were unable to detect physical interactions between these factors. Taken together, we find a lack of evidence for a functional complex containing the yeast CDK complexes and the U2 snRNP. These results suggest that if P-TEFb associates with Tat-SF1 and the U2 snRNA to stimulate transcription *in vivo*, these interactions are not conserved in yeast.

## Results

### Neither the Tat-SF1 yeast homolog *CUS2* nor the U2 snRNA exhibits genetic interactions with the yeast homologs of P-TEFb

Cus2 and the U2 snRNP interact to stabilize secondary structures of the U2 snRNA that are important for spliceosome assembly (Figure 1). In light of evidence that the mammalian Tat-SF1 interacts with snRNPs and has functional interactions with P-TEFb, we considered the possibility that Cus2 and U2 could also affect transcription via interactions with the P-TEFb homologs. To address this possibility, we began by examining genetic interactions between *CUS2* and transcription factors, particularly the putative P-TEFb homologs.

Synthetic interactions (such as synthetic lethality) are a hallmark behavior of genes involved in a common function [40]. Not surprisingly, *CUS2* deletion exhibits synthetic interactions with genes encoding other splicing factors including the U2 snRNA and several proteins associated with U2 snRNPs including Prp5 and Cus1 [16], [23], [41]. Similarly, deletion of components of the CDK complexes leads to severe synthetic growth defects when combined with deletion or mutation of factors involved in transcriptional elongation, including TFIIS, the Spt4/5 complex, and the CTD of RNAPII [33], [37], [39], [42]–[45]. We predicted that if the Bur or Ctk complexes shared a functional overlap with *CUS2*, as is the case with P-TEFb and Tat-SF1, we might observe synthetic mutant phenotypes when mutations in the Bur or Ctk complexes were combined with deletion of *CUS2*.

**Figure 2:**
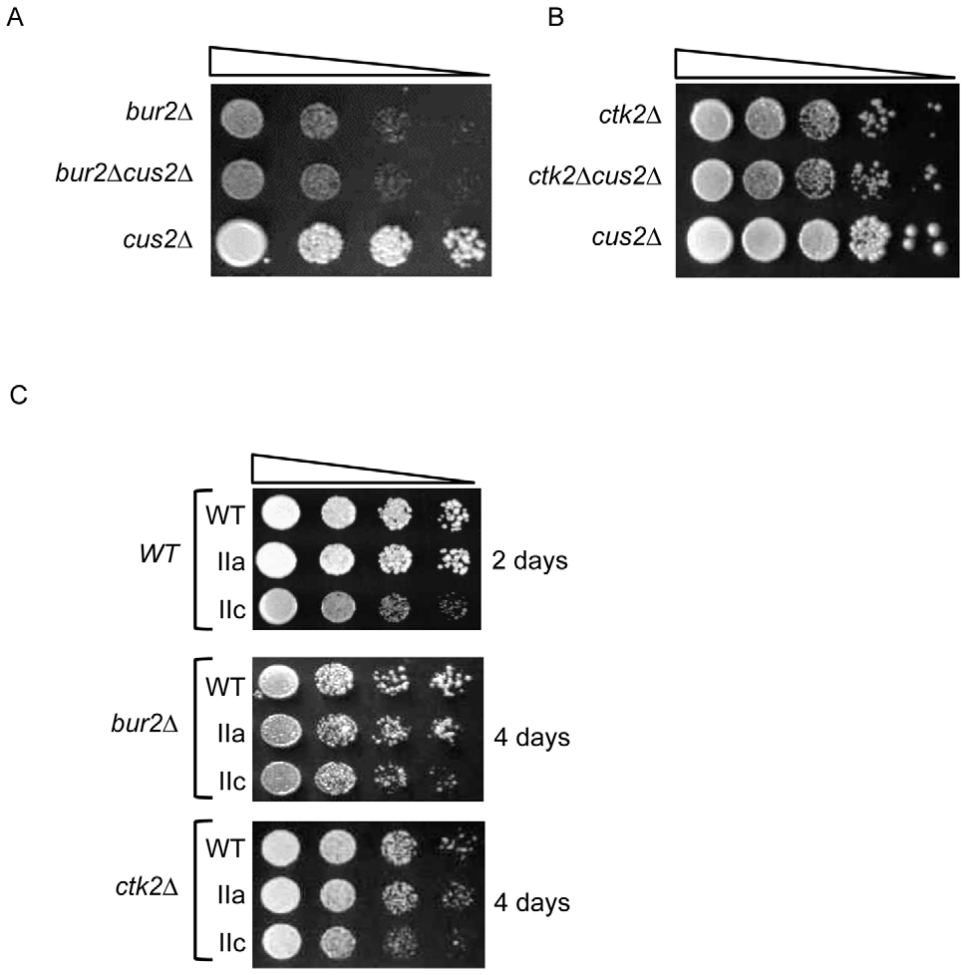
Neither Cus2 nor the U2 snRNA exhibits genetic interactions with p-TEFb-associated cyclin homologs. (A) Analysis of *bur2Δ cus2Δ* double mutant. The indicated strains were grown in YPD and four ten-fold serial dilutions were spotted onto YPD plates and grown at 30°C for 3 days. (B) Analysis of *ctk2Δ cus2Δ* double mutant. Cells were treated as described in (A). (C) Analysis of the U2 snRNA conformational mutants. Strains harboring the wild-type U2 snRNA (*URA3* plasmids, pRS316) and the indicated mutant snRNA (*LEU2* plasmids, pRS315) were grown in selective SC-ura-leu media and four ten-fold serial dilutions were spotted onto 5-FOA-Leu to select for loss of the wild-type U2 snRNA *URA3*-marked plasmid. Plates were grown at 30°C for the indicated number of days.

To address this possibility, we crossed a *cus2Δ* strain with both *bur2Δ* and *ctk2*Δ strains and then analyzed the double mutant progeny (Figure 2). *BUR2* and *CTK2* are both non-essential components of their P-TEFb-like complexes. Deletion of *BUR2* or *CTK2* eliminates the kinase activity of their respective complexes and results in phenotypes consistent with their roles in transcription [46], [47]. The growth rate of a *cus2Δ* mutant is similar to that of wild type cells; whereas a *bur2Δ* mutant shows a significant growth defect (Figure 2A). This slow growth phenotype is unchanged in the *cus2Δ bur2Δ*strain. Similarly, deletion of *CTK2* results in a slow growth phenotype that is unaffected by *CUS2* deletion (Figure 2B). Unlike other factors that contribute to Bur or Ctk activity and reveal this through synthetic interactions, we find no evidence of such a relationship between the CDKs and *CUS2*.

The ability of P-TEFb and Tat-SF1 to physically interact with the U snRNAs, particularly the U2 snRNA was shown to be crucial for the stimulatory effect on transcription. Since Cus2 has been shown to specifically associate with the U2-IIc conformation, we hypothesized that U2 snRNA mutants that preferentially form or disrupt this structure might play a functional role in transcription elongation and that this might be revealed by genetic interactions with the CDKs.

To determine whether Bur or Ctk complex function is influenced by a particular conformation of the U2 snRNA, *bur2Δ and ctk2Δ* strains were each crossed with a strain in which the genomic U2 (*SNR20*) gene was deleted and the U2 snRNA gene was harbored on a plasmid. Mutant U2 snRNA alleles were introduced to the resultant double mutant strains by plasmid shuffling. These previously characterized U2 snRNA plasmids encoded alleles that preferentially form either the U2-IIc or IIa conformation [16], [19]–[21], [23]–[25]. The U2-IIc allele consists of a G53 to A mutation that favors the U2-IIc conformation by abrogating the formation of the essential IIa stem-loop. The U2-IIa allele hyperstabilizes the essential IIa stem-loop element by deletion of the region of phylogenetically conserved complementarity to stem-loop IIa combined with conversion of the AU stem-pairs to more thermodynamically stable GC pairs (Figure 1B).

As previously reported, in a wild-type background, the U2-IIc allele confers a slow growth phenotype (Figure 2C) consistent with its defect in splicing (data not shown). However, the U2-IIa allele confers no growth defect (Figure 2C) or splicing defect (data not shown). Expression of the mutant U2-IIa or U2-IIc snRNA allele does not alter the growth of *bur2Δ* (Figure 2C). Similarly, the slow-growth phenotype conferred by *CTK2* deletion is not altered by the U2 structural mutants (Figure 2C). These data suggest that, like *CUS2*, neither the U2-IIc nor the U2-IIa conformation displays functional overlap with the Bur or Ctk complexes. Because of the orthologous relationship between the Bur complex and the P-TEFb complex, we looked closely at a variety of readouts associated with Bur complex function.

### Neither changes in Cus2 levels nor alteration of U2 snRNA conformation confer the transcription-related phenotypes of the P-TEFb homologs

Deletion of *BUR2* results in several phenotypes that are classic indicators of defects in transcription, including sensitivity to the drug 6-azauracil (6-AU) [46], [48]. Treatment of cells with 6-AU results in nucleotide depletion and enhances the requirement for a fully functioning transcription apparatus for efficient transcription [49]. Hence, genes encoding transcription elongation factors are often required for cell viability in the presence of 6-azauracil. If *CUS2* is involved in regulating transcription elongation through interactions with the Bur complex, then it is likely that *CUS2* will also exhibit this phenotype or affect the 6-AU sensitivity of *bur2* mutants.

**Figure 3:**
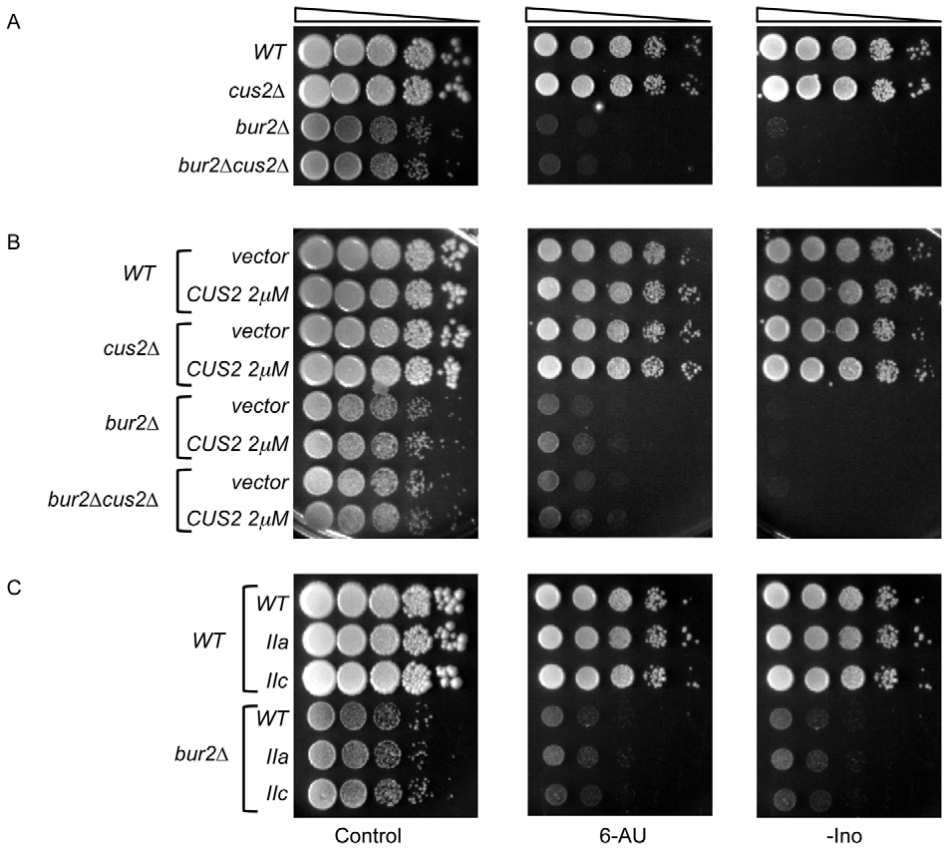
Phenotypic analysis of *bur2*Δ double mutant strains. (A) *CUS2* deletion does not confer sensitivity to 6-AU or inositol auxotrophy. Strains used in these studies were transformed with pRS316 (*URA3*) to allow for growth on media containing 6-azauracil (6-AU). Serial dilutions of the indicated strains were spotted onto solid media medium lacking uracil (Control), in the presence of 6-AU (100 µg/ml), or on media lacking inositol (-INO) and incubated at 30°C for 3–6 days. (B) *CUS2* overexpression does not confer 6-AU sensitivity, inositol auxotrophy, or suppress these phenotypes of *bur2*Δ. The indicated strains carrying *URA3*-marked plasmids (vector or *CUS2* on a 2μ plasmid) were grown in SC-uracil and four ten-fold serial dilutions were spotted onto solid media medium lacking uracil (Control), in the presence of 6-AU (100 µg/ml), or on media lacking inositol (-INO) and incubated at 30°C for 3–6 days. (C) U2 snRNA mutants do not exhibit sensitivity to 6-AU and are not auxotrophic for inositol. Mutant snRNAs were introduced into the indicated strains by plasmid shuffling. Strains were then re-transformed with pRS316 (*URA3*) and spotted onto the indicated media. Plates were grown at 30°C for the indicated number of 3–6 days.

To determine whether *CUS2* deletion confers 6-AU sensitivity or affects the 6-AU sensitivity of *bur2Δ*, we analyzed growth of *cus2Δ* and *cus2Δ bur2Δ* on media containing 6-AU. Deletion of *BUR2* alone results in sensitivity to 6-AU (Figure 3A). However, deletion of *CUS2* exhibits no 6-AU sensitive phenotype alone, nor does this mutation exacerbate the 6-AU sensitivity of *bur2Δ* cells as seen in the double mutant.

Because factors involved in the same pathways are sometimes able to compensate for one another when overexpressed [50] we considered the possibility that the Bur complex and *CUS2* acted in the same pathway such that deletion of one gene masked the phenotype associated with deletion of the other. To determine whether *CUS2* overexpression could compensate for the lack of *BUR2*, we generated strains in which *CUS2* was overexpressed on a high-copy 2μ plasmid. This allele was HA-tagged, and overexpression of the Cus2 protein was confirmed by western blot analysis using antibodies directed against the epitope (data not shown). *CUS2* overexpression does not affect the growth of the strains tested when compared to the vector alone (Figure 3B). Furthermore, overexpression of *CUS2* was not able to suppress the slow growth phenotype conferred by *BUR2* deletion. Next, we tested the ability of *CUS2* overexpression to suppress the 6-AU sensitivity of *BUR2* deletion. *CUS2* overexpression does not confer 6-AU sensitivity in wild type or *cus2Δ* cells nor does overexpression suppress the 6-AU sensitivity of *bur2Δ* cells (Figure 3B).

To determine whether particular U2 snRNA conformations exhibit sensitivity to 6-AU or affect *bur2Δ* 6-AU sensitivity, the double mutant strains were grown in the presence and absence of the drug (Figure 3C). In the *WT* background, neither the IIa nor the IIc U2 snRNA allele exhibits slow growth in the presence of 6-AU. Moreover, neither U2 snRNA mutant affected the 6-AU sensitivity of the double mutant. These data indicate that U2 snRNA is not able to compensate for loss of Bur2 in the presence 6-AU.

In addition to sensitivity to 6-AU, deletion of *BUR2* confers inositol auxotrophy, a phenotype that is often correlated with defects in transcription [46], [48], [51]. Mutation of particular components of the general transcription machinery including *BUR1* and *BUR2*, can lead to defects in the expression of *INO1*, the gene encoding inositol-1-phosphate synthase that converts glucose-6-phosphate into inositol. As a result, these mutant strains are unable to grow in the absence of inositol in the media.

To determine whether, like *BUR2*, *CUS2* or the U2 snRNA confers the Ino^−^ phenotype, the single mutant strains of each were grown on media lacking inositol. As previously described, the *bur2Δ* strain is unable to grow under these conditions (Figure 3). Unlike *bur2Δ*, strains in which *CUS2* is deleted or overexpressed grow similarly to the wild type strain in the absence of inositol and do not exhibit the Ino^−^ phenotype. Furthermore, neither deletion nor overexpression of *CUS2* alters the Ino^−^ phenotype of the double mutant *bur2Δ cus2Δ* strain. Similarly, the U2-IIa and U2-IIc mutants do not exhibit inositol auxotrophy, nor are they able to suppress this phenotype of the *bur2Δ* strain. Taken together, these data indicate that neither *CUS2* nor the U2 snRNA exhibit the 6-AU sensitivity or inositol auxotrophy shared by *BUR2* and other transcription factor mutant strains.

### The U2 snRNP does not exhibit genetic interactions with other general transcription factors

It is possible that the U2 snRNP may affect transcription but that *in vivo* this activity may only be revealed under certain conditions. The role for the Bur and Ctk complexes has been characterized, in part, by their genetic interactions with other components of the transcription apparatus. To address whether a U2 snRNP function in transcription can be revealed through functional interactions with elongation factors, we performed a targeted genetic screen between *CUS2* or the U2 snRNA and factors known to interact with the CDKs. Here, we looked for synthetic interactions between the splicing factors and the elongation factor *TFIIS* (*DST1*), which is synthetically lethal in combination with *bur2Δ* [52]; *SPT4* whose activity is regulated by the Bur complex [43]; and *LEO1*, a nonessential member of the PAF complex [52], [53] responsible for regulating histone modifications that promote active transcription. For comparison, we analyzed genetic interactions between the individual transcription elongation factors as well. The results of this screen along with previously published results are summarized in Table 1.

**Table 1:** Summary of phenotypic analysis of *cus2Δ* and U2 snRNA mutants compared to select transcription elongation factors.

	*bur2Δ*	*ctk2Δ*	*ctk3Δ*	*cus2Δ*	*U2 IIa*	*U2 IIc*
**Haploid growth**	++	+++	+++	++++	++++	+++
**6-Azauracil**	−	ND, *ctk1Δ* 6-AU^s^ [71]	ND, *ctk1Δ* 6-AU^s^ [71]	++++	++++	+++
**Bypass UAS**	Yes [61]	No	No	No	No	No
**GENETIC INTERACTIONS**						
***bur2Δ***						
***ckt2Δ***	SL					
***ctk3Δ***	SL	SGD				
***cus2Δ***	No SGD	No SGD	No SGD			
***U2 IIa***	No SGD	No SGD	No SGD	No SGD		
***U2 IIc***	No SGD	No SGD	No SGD	SL	ND	
**CTD truncation**	ND, *bur1-2* SGD [45]	ND, *ctk1Δ* SGD [45]	ND, *ctk1Δ* SGD [45]	No SGD	ND	ND
***dst1Δ***	SGD [43]	SGD	SGD [72]	No SGD	No SGD	No SGD
***leo1Δ***	SGD [35]	SGD [72]	ND, *ctk1Δ*SGD [53], [72]	ND	No SGD	No SGD
***spt4Δ***	SL [52]	ND, *ctk1Δ*SGD [43]	ND, *ctk1Δ* SGD [43]	ND	No SGD	No SGD

SGD, Synthetic Growth Defect; SL, Synthetically lethal; 6-AU^s^, 6-azauracil sensitive; ND, Not Determined.

Deletion of *BUR2* is synthetically lethal in combination with deletion of either *CTK2* or *CTK3*, a fact that highlights the high degree of functional overlap between these complexes. Both the Bur and Ctk complexes are dependent upon an intact polymerase CTD (Rpb1), as mutations in the components of either CDK have previously been shown to be inviable in combination with *rpb1* truncation alleles [43], [45]. Similarly, combining *dst1Δ* with mutations in the CDKs causes a severe synthetic growth defect. In contrast, neither the *rpb1* CTD truncations combined with *CUS2* deletion nor the *dst1Δ cus2Δ* double mutants display synthetic growth defects (Table 1). Similarly, the U2 snRNA mutants we tested did not display interactions with *DST1*.

In addition to their roles in phosphorylation of the CTD of RNA polymerase, the Bur and Ctk complexes regulate transcription in part through interactions with the Spt4-Spt5 complex. Spt4-Spt5 complex is conserved across eukaryotes and the mammalian counterparts interact with P-TEFb [54]. Both *SPT4* and *SPT5* are required for efficient transcription elongation and have been implicated as having roles in coupling transcription and splicing [55]. *SPT4* exhibits genetic interactions with *BUR2*, *CTK2*, *DST1* and the polymerase [43], [52]. However, unlike these known transcription factors, we find no similar interaction between *SPT4* and either deletion of *CUS2* or its overexpression.

Leo1 is a component of the PAF elongation complex that regulates transcription elongation via its role in histone modification. *LEO1* and other PAF subunits exhibit interactions with both the Bur and the Ctk complex [35], [44], [52], [53], [56]–[58]. Conversely, we found that *leo1*Δ cells exhibited no synthetic growth defect when combined with the U2 snRNA alleles.

As is the case with *bur2Δ* cells, deletion of *DST1*, *LEO1*, or *SPT4* renders cells sensitive to 6-AU, thus indicating their roles in regulating transcription [53], [59], [60]. To determine whether U2 snRNA alleles affect the 6-AU sensitivity of these strains we tested the growth of the double mutants on medium containing 6-AU. Alterations in the U2 snRNA secondary structure failed to produce significant changes in the 6-AU sensitivity of these strains (data not shown and Table 1). Taken together, the genetic analyses summarized in Table 1 suggest that neither *CUS2* nor the U2 snRNA conformation mutants exhibit functional overlap with transcription factors that interact with the CDK complexes.

### Neither changes in Cus2 levels nor alteration of U2 snRNA conformation exhibits Bur^−^ or Spt^−^ phenotypes

*BUR1* and *BUR2* were initially identified in a screen for mutants that exhibited increased transcription of the *SUC2* gene in the absence of its upstream activating sequence (UAS) [61]. Deletion of the *SUC2* upstream activating sequence (*suc2*Δuas) abolishes transcription of this gene and, as a result, prevents growth on media containing sucrose as the carbon source. Mutations that bypass the UAS requirement exhibit the Bur^−^ phenotype and increase transcription from *suc2*Δuas [48]. We hypothesized that if the U2 snRNP components are involved in Bur complex activity at core promoters, mutations in *CUS2* or U2 snRNA should also exhibit the Bur^−^ phenotype. To test this hypothesis, we generated double mutant strains harboring the *suc2*Δuas mutation and *CUS2* deletion or the *SNR20* deletion complemented by a U2 snRNA allele on a plasmid. The resulting strains were tested for their ability to grow on sucrose plates. The *bur2-1* mutant strain was able to grow on sucrose plates as previously reported, while the *cus2*Δ, *CUS2* overexpressing, U2-IIa and U2-IIc snRNA mutant strains each failed to grow on sucrose plates (Table 1). These results suggest that, unlike mutations in *BUR2*, these splicing factor mutants cannot bypass the requirement for the UAS for transcription.

The Suppressor of Ty (*SPT*) genes were originally identified by their ability to genetically suppress the transcriptional defects conferred by Ty element insertion [61]. Strains harboring a *δ* Ty element in the *HIS4* gene promoter are His^−^ but in the presence of *spt* mutations become His^+^. The *spt* mutants suppress transcriptional defects through several different mechanisms, all involving alteration of transcription. Mutations in *SPT4*, *SPT5*, *BUR1* and *BUR2* confer Spt^−^ phenotypes indicative of their roles in regulating transcription events [60], [61]. Specific mutations in either component of the Bur complex have been found to exhibit Spt^−^ phenotypes [33], [45], [46], [48], [62]. For example, the *bur2-1* mutant is Spt^−^ and overexpression of the *bur1-3* allele exhibits a dominant negative Spt^−^ phenotype. If the U2 snRNP exhibits functional overlap with the Bur complex, then *CUS2* or the U2 snRNA may also confer the Spt^−^ phenotype.

**Figure 4:**
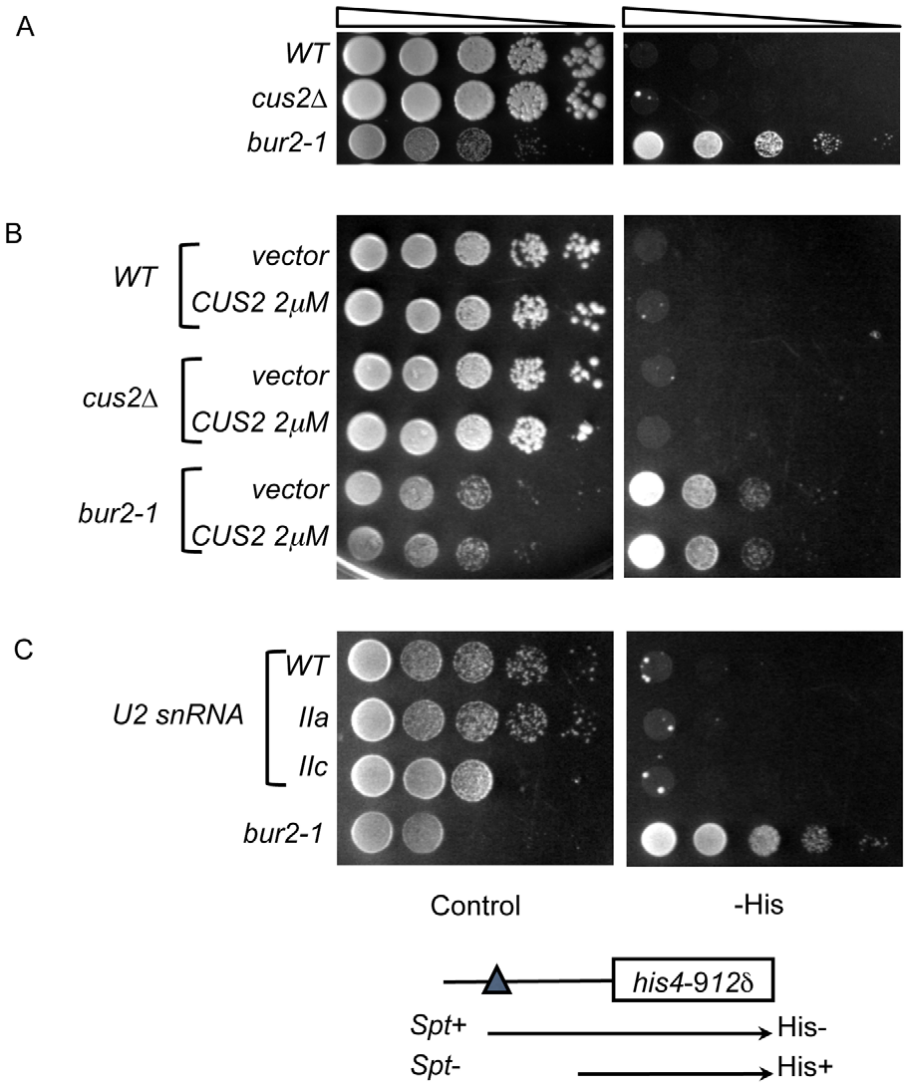
Neither Cus2 nor the U2 snRNA exhibits an Spt^**−**^ phenotype. The strains used in this study all contain the *his4-912δ* mutation. *bur2-1* has been previously characterized as Spt^−^ (A) The indicated strains were grown in YPD and four ten-fold serial dilutions spotted onto media containing (Control) or lacking histidine (-His) and grown at 30°C for 5 days. (B) The indicated strains carrying *URA3*-marked plasmids (vector or *CUS2* on a 2μ plasmid) were grown in SC-uracil and four ten-fold serial dilutions were spotted onto SC-uracil (Control) or onto media lacking uracil and histidine (-His) and incubated at 30°C for 5 days. (C) Mutant snRNAs were introduced into the indicated strains by plasmid shuffling. Strains were grown overnight in SC-leu medium and plated onto SC-leu (Control) or SC-leu-his (-His) media. Plates were grown at 30°C for 5 days.

To test this we generated double mutant strains of these factors and the *his4-912δ* mutant. The resultant double mutants were assayed for their ability to grow on plates lacking histidine. As previously described, the *bur2-1* mutant suppresses the *his4-912δ* ty insertion mutation and allows for growth on media lacking histidine (Figure 4). Conversely, neither *CUS2* deletion (Figure 4A) nor overexpression (Figure 4B) restored growth on media lacking histidine. Similarly, the U2 snRNA mutants did not grow on media lacking histidine and did not exhibit an Spt^−^ phenotype similar to mutations in *BUR2* (Figure 4C). These studies suggest that, unlike the Bur complex, these splicing factors are unable to affect transcription from promoters disrupted by a Ty insertion.

### Neither Cus2 nor U2 snRNA exhibits physical interactions with the Bur complex

The mammalian study of the role of Tat-SF1 in *in vitro* transcription demonstrated that Tat-SF1 and accompanying U2 snRNP associated with P-TEFb via interactions with its cyclin subunit T1 [1]. If a parallel interaction exists in yeast, then we would expect to detect physical interactions between the homologous cyclin Bur2 and both Cus2 and U2 snRNA.

To determine whether Bur2 physically interacts with Cus2 or the U2 snRNA we performed co-immunoprecipitation experiments. Co-immunoprecipitations were performed using TAP-tagged Bur2 strains in which a 6×His epitope-tagged Cus2 is expressed from the *GAL1* promoter on a plasmid, and Cus2 production was induced by growth in galactose for three hours. This allowed us to enrich for the Cus2 protein available for immunoprecipitation or co-immunoprecipitatation. Under these conditions Cus2 overexpression produced no detectable phenotype and complementation tests have shown that the 6×His epitope did not interfere with the function of Cus2 (data not shown and personal communication, M. Ares). We have demonstrated that Bur1 and Bur2 co-immunoprecipitate (data not shown) indicating that the Bur2 pulldown used in these studies represents pulldown of both factors. Parallel Cus2-His or Bur2-TAP immunoprecipitates were split and examined for protein interactions with Bur2 or Cus2, respectively, by western and for protein-RNA interactions by primer extension. Initial experiments failed to detect an interaction between Bur2 and either the U2 snRNA or Cus2. However, if an *in vivo* interaction exists but is weak, transient, or occurs relatively infrequently, the interaction may be difficult to detect using this method. To address this possibility, we used *in vivo* formaldehyde cross-linking to enrich for weak interactions.

**Figure 5:**
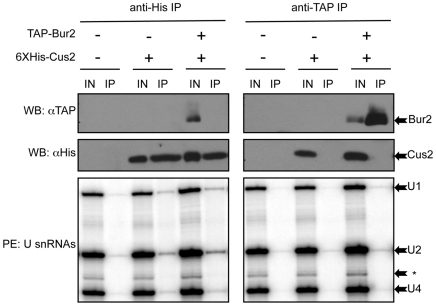
Neither Cus2 nor the U2 snRNA co-immunoprecipitate the Bur complex. Anti-His and anti-TAP immunoprecipitations were performed on whole cell lysates made from formaldehyde cross-linked Bur2-TAP tagged strains carrying a vector or a 6×His tagged *GAL1*-*CUS2* plasmid grown in galactose media. The presence or absence of the epitope tag is denoted by a “+” or “−,” respectively. “IN” indicates the input sample, “IP” indicates the immunoprecipitate. Samples were split and analyzed in parallel for protein interactions by western blotting (WB) and for protein-RNA interactions by primer extension (PE). In the upper panel, the precipitates were blotted with anti-TAP antibody for detection of the Bur2 protein (indicated by the arrow). In the middle panel, precipitates were blotted with anti-His antibody for detection of Cus2 protein. In the bottom panel, total RNA from the precipitates was extracted and probed by primer extension for the presence of U snRNAs using oligos specific to U1, U2, and U4 snRNAs whose products are indicated by arrows. “*” indicates a non-specific product of the primer extension reaction.

As expected, we observe physical interactions between Cus2 and the U2 snRNA in the anti-His precipitates (Figure 5). This interaction is specific for the U2 snRNA, although we detect some U1 snRNA that may represent an interaction with Cus2 and the prespliceosome. Although Cus2 shows positive interactions with these spliceosomal snRNAs, we failed to detect the Bur2 protein in these precipitates. Similarly, neither the Cus2 protein nor U2 snRNA were detected in the Bur2-TAP immunoprecipitate. These data suggest that, although Cus2 is able to interact with the U2 snRNA under the conditions tested, these interactions do not occur in the context of a larger complex containing the Bur complex and are therefore distinct from those interactions reported in metazoans.

## Discussion

Previous studies have suggested that transcription and splicing are functionally coupled. A key prediction of this model is that specific transcription factors and splicing factors physically interact and that these interactions would affect splicing, transcription, or both. Mammalian studies have demonstrated such a relationship between the transcriptional elongation factors P-TEFb and Tat-SF1 [1]. The experiments described here set out to determine whether the yeast homologs of P-TEFb and components of the U2 snRNP have similar physical and functional interactions. We find that the U2 snRNP components do not affect transcription in a detectable way. Furthermore, we were unable to detect physical interactions between the U2 snRNP components and the P-TEFb homologs. Taken together, we find a lack of evidence for a functional interaction between the P-TEFb homologs and the yeast U2 snRNP, particularly in transcription. Our findings do not eliminate the possibility that the U2 snRNP plays some role in transcription, perhaps of specific genes under specific conditions not tested in this study. However, if such a role exists, it does not appear to generally involve interactions with the Bur or Ctk complexes or their characterized roles in transcription.

Although there were already suggestions in the literature that transcription and, in particular, the CTD of RNAPII could affect splicing, the work by Fong & Zhou was the first to demonstrate that specific interactions between the splicing and transcription machineries led to a reciprocal relationship between transcription and splicing. These *in vitro* studies suggested that a similar interaction occurs *in vivo*, perhaps to couple transcription and splicing. However, a parallel *in vivo* relationship between the core splicing machinery—namely the U2 snRNP—and P-TEFb has not yet been established. Interestingly, the SR protein SC35 does facilitate P-TEFb recruitment to specific genes *in vivo*, and SC35 depletion is associated with reduced CTD Ser2 and defective transcription elongation [12].

Tat-SF1 has been implicated in both viral transcription and splicing, as well as in general elongation [63]–[65]. However, the *in vivo* role of Tat-SF1 remains to be fully elucidated. Several *in vitro* studies have suggested a role for Tat-SF1 in HIV-1 Tat-transactivation [63], [64]. Furthermore, *in vivo* data has suggested instead that Tat-SF1 has important post-transcriptional roles in regulating viral RNAs. In fact, one recent report suggests that Tat-SF1 affects HIV-1 replication by regulating the splicing, but not the transcription, of viral transcripts, while another demonstrates that Tat's roles in HIV1 transcription and splicing are functionally uncoupled and that Tat-SF1 facilitates Tat's role in the splicing of viral transcripts [66], [67]. Moreover, Tat-SF1 was shown to facilitate influenza replication via its role in viral packaging, as opposed to having a role in viral RNA synthesis [17]. Taken together, these *in vivo* studies demonstrate that the important cellular role of Tat-SF1 includes, and may primarily in post-transcriptional regulation of RNAs, like splicing. Indeed, Tat-SF1, along with other transcription factors, has been detected in some complexes containing splicing factors [68], and Tat-SF1 co-immunoprecipitates with the splicing factor SF3a66 [16]. A parallel relationship exists between Cus2 and Prp11, the yeast homolog of SF3a66, and suggests that Tat-SF1 may have roles in splicing similar to those of Cus2 [16]. Future studies will be needed to determine whether Tat-SF1, like Cus2, has a role in regulating the splicing of endogenous genes.

### Is the ability of Tat-SF1 to stimulate viral transcription evolutionarily conserved?

Although we were unable to demonstrate a clear role for Cus2 in regulating transcription, it is possible that through evolution P-TEFb and Tat-SF1 have acquired additional functions that allow them to associate in a novel way not seen in yeast. Although Tat-SF1 and *CUS2* are closely related and contain two highly similar RRMs, the extensive C-terminal acidic domain of Tat-SF1 is greatly reduced in Cus2. It is possible that this longer acidic has allowed Tat-SF1 to acquire a new functions absent in its yeast counterpart. The acidic domain of Tat-SF1 is required to facilitate the protein's association with P-TEFb and for efficient transactivation of a TAR-containing reporter. In yeast, this portion of the Cus2 protein is important for its function in U2 snRNA folding as deletion of this region results in reduced function toward misfolded U2 snRNA [16]. The extended acidic domain of Tat-SF1 is therefore an attractive candidate for the region of the protein that coordinates splicing and transcription.

Interestingly, a recent report identified Cus2 as an important factor for influenza virus RNA synthesis in infected yeast cells [17]. Here, yeast cells supporting the replication and transcription of the influenza genome demonstrated lowered viral RNA expression in the absence of *CUS2*. This effect was recapitulated with siRNA-mediated knockdown of Tat-SF1 in influenza infected HeLa cells due to a direct role for Tat-SF1 in facilitating assembly of viral RNPs that are essential for downstream viral RNA synthesis. These data suggest that viral machineries are able to utilize Cus2 and Tat-SF1 in a similar manner through recognition of homologous portions of these proteins rather than the extended C-terminal acidic domain. It is clear that at least in some cases, viruses utilize host factors in ways unique to viral infection. It will be interesting to determine whether the effects of Cus2 and Tat-SF1 on influenza replication are extended to other viruses like HIV1.

In summary, the data reported here are inconsistent with a general role for the U2 snRNP in regulating transcription in yeast, but do not preclude the idea that viruses are able to utilize splicing factors in novel ways to facilitate viral replication. Future studies will be needed to determine whether Tat-SF1 plays a role in coupling splicing and transcription of endogenous genes and to clarify the precise conditions under which this coupling occurs.

## Materials and Methods

### Yeast strains and growth

The strains used in this study are listed in Table S1 in Supporting Information S1 and are in the BY4743 strain background with the exception of the *spt* and *bur* mutant strains, provided by Karen Arndt and Gregory Prelich. Strains containing multiple disruptions with the same auxotrophic marker were obtained from genetic crosses and confirmed by PCR. All strains were propagated according to standard procedures in the appropriate selective media. Plasmid shuffling was performed on selective 5- fluoroorotic acid (5-FOA) plates. Sucrose plates prepared as described elsewhere [69] and supplemented with 1 µg/ml antimycin A. Inositol media were prepared from a yeast nitrogen base containing ammonium sulfate but lacking inositol (QBIOgene) and supplemented with the appropriate nutrients; inositol was added to 100 µM where indicated. Standard methods for transformations and media preparation were used as described in Methods in Yeast Genetics: A Cold Spring Harbor Laboratory Course Manual. The plasmids used in this study are listed in Table S2 in Supporting Information S1.

### Viability assay/dilution series

For growth analysis, strains were grown overnight in the appropriate selective media at 30°C. Cells were diluted and incubated at 30°C until all strains reached the same O.D._600_ (between 0.3–0.5). A ten-fold serial dilution of each strain was spotted onto the proper selective plates and incubated for the indicated number of days.

### 6-azauracil plate assay

The yeast strains used in this assay were transformed with the *URA3* CEN plasmid pRS316 or a *URA3*+ 2μ plasmid, and selected on synthetic complete media lacking uracil. The transformed colonies were grown in SC-uracil liquid media up to O.D._600_ of approximately 0.5, serially diluted 10-fold onto SC-uracil plates with or without 100 µg/ml 6-azauracil. Plates were incubated at 30°C for 3–6 days.

### Cross-linking co-immunoprecipitation

Protein and RNA co-immunoprecipitation experiments were carried out as described in [70] with the following modifications. Cells were grown in SC-uracil 2% raffinose to an O.D._600_ of approximately 0.4. Cus2 expression was induced by the addition of galactose to a final concentration of 2% and incubated for an additional 3 hours. Cells were cross-linked for 15 minutes with formaldehyde to a final concentration of 1%. The cells were then harvested by centrifugation, washed, and lysed by vortexing with glass beads in cold lysis buffer (50 mM HEPES-KOH pH 7.5, 140 mM NaCl, 1 mM EDTA pH 8.0, 1% Triton-X, 0.1% Deoxycholate; Roche Complete protease inhibitor, and 40U Promega RNasin). Pre-cleared whole cell extracts were incubated for 2.5 hours at 4°C with IgG Sepharose 6 Fast Flow beads (GE Healthcare) or NiNTA beads (Qiagen) as appropriate. Beads were washed as previously described and divided into two samples for subsequent protein or RNA analysis. For protein analysis, the bound protein was eluted by boiling the beads for 10 minutes in SDS-PAGE sample buffer. Samples were fractionated by SDS-PAGE electrophoresis and transferred to a nitrocellulose membrane for immunoblotting with 1∶3000 dilution of anti-TAP (Upstate 12–342) and 1∶5000 dilution of anti-His (Santa Cruz Biotechnology 804), followed by chemiluminescent detection (Pierce). For RNA analysis, the co-immunoprecipitated RNA was eluted as described by Selth et al., isolated by phenol chloroform extraction, and ethanol precipitated. The precipitated RNA was used as a template for primer extension using oligomers complementary to the U1, U2, and U4 snRNAs. The sequences of these primers are listed in Table S3 in Supporting Information S1.

## Supporting Information

Supporting Information S1 (DOC)
