# Supplementary material for: An Investigation of a Role for U2 snRNP Spliceosomal Components in Regulating Transcription
Source: PLoS One. 2011 Jan 24;6(1):e16077. doi: 10.1371/journal.pone.0016077 (PMC3025917; doi:10.1371/journal.pone.0016077)
Supplement: Supporting Information S1 — (DOC) [file pone.0016077.s001.doc]

**Supporting Information**

**Table S1** List of yeast strains used in this study

**Table S2** List of plasmids used in this study

**Table S3** List of U snRNA oligomers used for primer extension

# Table S1. Strains used in this study

| **Strain** | **Genotype** | **Reference** |
| --- | --- | --- |
| WT | *MATα ura3∆0 leu2∆0 his3∆1 lys2∆0* | This study |
| *bur2∆* | *MATa ura3∆0 leu2∆0 his3∆1 met15∆0 bur2::kanMX4* | This study |
| *cus2∆* | *MATα ura3∆0 leu2∆0 his3∆1 lys2∆0 cus2::kanMX4* | This study |
| *ctk2∆* | *MATa ura3∆0 leu2∆0 his3∆1 met15∆0 ctk2::kanMX4* | This study |
| *bur2∆* *cus2∆* | *MAT*a *ura3∆0 leu2∆0 his3∆1 met15∆0 bur2*::*kanMX4* *cus2*::*kanMX4* | This study |
| *ctk2*∆ *cus2*∆ | *MAT*a *ura3*∆*0 leu2*∆*0 his3*∆*1 met15*∆*0 ctk2::kanMX4* *cus2*::*kanMX4* | This study |
| KY616 | *MAT*a *his4-912*∆δ *lys2-128*∆δ *leu2*∆*1* *ura3-52* *suc2*∆*uas*(-1900/-390) | [1] |
| GY103 | (*MAT*a *his4-912*∆δ *lys2-128*∆δ *suc2*∆*uas(*-*1900/*-*390) ura3-52 trp1*∆*63 bur2-1* | [2] |
| KY616 U2∆+pAB146 | *MAT*a *his4-912*∆δ *lys2-128*∆δ *leu2*∆*1 ura3-52 suc2*∆*uas*(-1900/-390) *U2::HIS3* [pAB146] | This study |
| U2∆+pAB146 | *MAT***α** *ura3*∆*0 leu2*∆*0 his3*∆*1 lys2*∆*0 U2::HIS3* [pAB146] | This study |
| *bur2*∆ U2∆+pAB146 | *MAT*a *ura3*∆*0 leu2*∆*0 his3*∆*1 met15*∆*0* *bur2*::*kanMX4* *U2::HIS3* [pAB146] | This study |
| *ctk2*∆U2∆+pAB146 | *MAT*a *ura3*∆*0 leu2*∆*0 his3*∆*1 met15*∆*0 ctk2*::*kanMX4* *U2::HIS3* [pAB146] | This study |
| *leo1*∆ U2∆+pAB146 | *MAT*α *ura3*∆*0 leu2*∆*0 his3*∆*1 lys2*∆*0 leo1*::*kanMX4* *U2::HIS3* [pAB146] | This study |
| *spt4*∆ U2∆+pAB146 | *MAT*α *ura3*∆*0 leu2*∆*0 his3*∆*1 lys2*∆*0 spt4*::*kanMX4* *U2::HIS3* [pAB146] |  |
| *dst1*∆ U2∆+pAB146 | *MAT*α *ura3*∆*0 leu2*∆*0 his3*∆*1 lys2*∆*0 dst1::kanMX4* *U2::HIS3* [pAB146] | This study |
| Bur2-TAP | *MAT*a *BUR2-TAP:HIS3 ura3*∆*0 leu2*∆*0 his3*∆*1 met15*∆*0* | OpenBiosystems |

# Table S2 List of plasmids used in this study

| **Plasmid Number** | **Plasmid Description** | **Plasmid backbone** | **Reference** |
| --- | --- | --- | --- |
| pTAGCus2 | *Cus2-HA-6XHIS GAL1* promoter | pTAG | [3] |
| pTAG | *HA-6XHIS* | pYES 1.2 | [3] |
| Cus2pTAGnP | *Cus2-HA-6XHIS* native promoter | pTAG | [3] |
| pAB146 | *WT U2* | pRS316 | This study |
| pAB131 | *WT U2* | pRS315 | This study |
| pAB138 | *U2-IIa ∆CC/GC stem* | pRS315 | This study |
| pAB143 | *U2-IIc G53A* | pRS315 | This study |

**Table S3. List of primers used in this study**

| U1 snRNA | 5’ GAATGGAAACGTCAGCAAACAC 3’ |
| --- | --- |
| U2 430R | 5’ caaaaaatgtgtattg 3’ |
| U4 snRNA | 5’ ACCATGAGGAGACGGTCTGG 3’ |

**REFERENCES**

1. Costa P, Arndt K (2000) Synthetic lethal interactions suggest a role for the Saccharomyces cerevisiae Rtf1 protein in transcription elongation. Genetics 156: 535-547.

2. Yao S, Neiman A, Prelich G (2000) BUR1 and BUR2 encode a divergent cyclin-dependent kinase-cyclin complex important for transcription in vivo. Mol Cell Biol 20: 7080-7087.

3. Yan D, Perriman R, Igel H, Howe KJ, Neville M, et al. (1998) CUS2, a yeast homolog of human Tat-SF1, rescues function of misfolded U2 through an unusual RNA recognition motif. Mol Cell Biol 18: 5000-5009.
